# A Quantitative Study on Anonymity and Professionalism within an Online Free Open Access Medical Education Community

**DOI:** 10.7759/cureus.788

**Published:** 2016-09-18

**Authors:** Daneilla Dimitri, Andrea Gubert, Amanda B Miller, Brent Thoma, Teresa Chan

**Affiliations:** 1 Family Medicine, McMaster University; 2 Resident, McMaster University; 3 Emergency Medicine, Regions Hospital; 4 College of Medicine, University of Saskatchewan; 5 Faculty of Health Sciences, Division of Emergency Medicine, McMaster University

**Keywords:** anonymity, professionalism, social media, free open access medical education, online discussion

## Abstract

The increasing use of social media to share knowledge in medical education has led to concerns about the professionalism of online medical learners and physicians. However, there is a lack of research on the behavior of professionals within open online discussions. In 2013, the Academic Life in Emergency Medicine website (ALiEM.com) launched a series of moderated online case discussions that provided an opportunity to explore the relationship between anonymity and professionalism.

Comments from 12 case discussions conducted over a one-year period were analyzed using modified scales of anonymity and professionalism derived by Kilner and Hoadley. Descriptive statistics and Spearman calculations were conducted for the professionalism score, anonymity score, and level of participation. No correlation was found between professionalism and anonymity scores (rho = -0.004, p = 0.97). However, the number of comments (rho = 0.35, p < 0.01) and number of cases contributed to (rho = 0.26, p < 0.05) correlated positively with clear identification.

Our results differed from previous literature, the majority of which found anonymity associated with unprofessionalism. We believe that this may be a result of the fostering of a professional environment through the use of a website with a positive reputation, the modelling of respectful behaviour by the moderators, the norms of the broader online community, and the pre-specified objectives for each discussion.

## Introduction

Social media, defined as the creation and exchange of user-generated content via virtual networks and communities using internet applications, is increasingly being used to share knowledge in medical education [1]. The FOAM (‘Free Open-Access Medical Education’) movement has led to a rapid growth in the use of blogs and podcasts as a delivery mechanism for online medical education in emergency medicine [1-2]. Educators and learners have turned to platforms, such as Twitter, to create online communities of practice [3-8].

With the rise of social media in medical education, concerns have been raised about the professionalism of the learners using these platforms. The literature contains review papers on social media, but these papers often focus on the negative effects of social media usage by learners [9-16]. Of note, Byrnolf, et al. investigated the potential breaches of medical ethics committed by physicians and medical students on Twitter and found that unprofessionalism was more common among users writing under pseudonyms than recorded names [9]. Although their study found a correlation between unprofessionalism and anonymity, its analysis failed to distinguish between personal and professional personae and the results may have been skewed by personal accounts that were not intended to be part of the professional sphere. There is a lack of literature on professionalism in open, online discussions that are conducted in professional environments.

Studies regarding professionalism and online behavior are inconsistent. One study found that pseudonymity, defined as the use of aliases, can play a valuable role in online learning and increase participation [17]. A commonly cited theory, ‘the online disinhibition effect’, has found that increasing anonymity using an online persona can lead to disinhibition that is either benign or toxic [14, 18-19]. Some people disclose personal information and demonstrate unusual kindness and generosity while others are critical, rude, and threatening.

In 2013, the ALiEM.com launched a series of online cases that prompted a global discussion (The Medical Education in Cases Series) [20]. Each month, a case is launched from the blog to which users can reply and discuss in the comments section. The cases focus on topics, such as ethics, education, and professionalism. These monthly discussions have led to the development of a community of practice that includes core members (ALiEM team members who act as moderators), active members (frequent commenters), and peripheral participants (infrequent commenters, readers). Case discussions are held openly on the ALiEM.com website, and interactions between participants who are using social media for medical education or faculty development can be publicly observed.

This study was designed to determine if there is a relationship between anonymity and professionalism within this online community of practice. We hypothesize a clear link between professional posts and identifiable individuals, while unprofessional posts are suspected to be generated by anonymous individuals. Additionally, this study should help us determine if a relationship exists between anonymity and participation. We hypothesize an increased rate of participation associated with anonymity. 

## Materials and methods

### Study population

The study population consisted of participants who contributed to the ALiEM Medical Education in Cases (MEdIC) Series discussions on the Academic Life in Emergency Medicine website between September 2013 and August 2014. This study was granted an exemption by the Hamilton Integrated Research Ethics Board since the data was already open and freely available online.

### Scoring

Scales derived and tested within a military population by Kilner and Hoadley were modified and used to assess anonymity and professionalism [21]. Anonymity was defined using a four-level scale that quantified the degree to which a user could be identified based on the information presented within their comment (Table 1). 

**Table 1:** Coding Rubric for Anonymity of Discussion Comments

Level of Anonymity	Description	Example Identifier
1	Anonymous	Anonymous
2	Username only (can include first or last name)	Njoshi8, Hans, RP
3	Username that indicates full name	Danica K
4	Username is probably real full name and/or they have other clear identifiers, i.e. picture, credentials	Allan McDougall

Professionalism was defined using a four-level scale that quantified the tone and contribution of the comment to the overall discussion (Table 2). The definitions for the levels of the anonymity and professionalism scales were modified according to agreed-upon descriptions by the three raters that fit the online medical education environment currently studied. The criteria for the highest level of professionalism (“Very positive: include multiple positive criteria”) was not well-defined in the original study, and as such, our team refined for our purposes to specify the positive contributions.

**Table 2:** Coding Rubric for Professionalism of Discussion Comments ^1 ^These comments were used as examples from Kilner and Hoadley’s study [21]. The fourth criteria (“Very positive: include multiple positive criteria”) was not well defined in the original study and was refined for our purposes to specify the positive contributions [21].

Level of Professionalism of Comment	Description	Example
1	Very negative: demeans with vulgarity	Get lost you dumbsh*t!^1^
2	Negative: critical of another, or cynical	You’re as screwed up as the Healthcare system.^1^
3	Positive: supportive of another	Great comment! I agree with…
4	Very positive: include multiple positive criteria, including the contribution of new ideas, resources, and discussion points.	In an age of evidence-based medicine now, we are also noting that our patients may want different things from us... What Doctors 'want'/think (sort of older, 2004 newspaper article): http://news.bbc.co.uk/2/hi/health/3706783.stm

Each comment was independently rated by three raters. Prior to rating, a calibration exercise was performed by the three raters. One case discussion was individually rated by the three investigators who then compared results, allowing for modification of scales where necessary. Following calibration, all comments made by discussion participants, but not moderators, were rated independently by all three raters.

### Statistics

To determine the extent of agreement between raters, intraclass correlation coefficients (ICC) were calculated for the results from each scale. Median scores for each comment were subsequently determined. Spearman correlations were calculated between professionalism scores, anonymity scores, and level of participation (as measured by the number of cases each participant contributed to and the number of comments that they made).

## Results

The demographics of the MEdIC Series discussion website can be seen in Table 3.

**Table 3:** MEdIC Series Website Visitor Demographics Between September 2013 to August 2014 ^1^ Interquartile range - IQR

MEdIC Series Website Visitor Demographics Between September 2013 to August 2014	
Number of views of the ALiEM.com website per year	1.2 million
Number of views per case within the first two weeks	1,000
Total number of participants across all cases	76
Median number of participants per case	Median (IQR )
Median number of comments per participant	1 (IQR^1^ 1-8)
Total number of moderators across all cases	4
Median number of cases moderators participated in	7.5 (IQR^1^ 4.75-12)

There were 338 comments made by 80 individuals, including moderators, on 12 cases between September 2013 and August 2014. The four moderators, with an average professionalism score of three out of four, functioned as hosts and seldom contributed actively to the discussion. Therefore, they were analyzed as a separate group, distinct from the participants-at-large.

After initial calibration, the ICC for the three raters were moderate-to-high, 0.81 (p < 0.001) for anonymity and 0.57 (p < 0.001) for professionalism. Subsequently, all cases from September 2013 to August 2014 were scored by the three raters and the median scores were used for the subsequent correlation statistics.

For non-moderator participants, the median professionalism score was four (interquartile range (IQR) 3-4) out of four and the median anonymity score was four (IQR 3-4) out of four for participants. Table 2 shows the raw percentage number of the comment quality in each month. There was no correlation between the participant’s median professionalism and anonymity scores (rho = -0.004, p = 0.97).

On secondary analysis, we assessed the relationship between anonymity and professionalism with the extent of participation. There was a moderate correlation between both participants’ identifiability (as indicated by a high score) and the number of comments they made (rho = 0.35, p <0.01) as well as the number of cases that they participated in (rho = 0.26, p < 0.05). Those who were more identifiable commented more. Professionalism also correlated with the number of cases participated in (rho = 0.27, p < 0.05) but not the number of comments that they made (rho = 0.076, p = 0.52).

## Discussion

The findings in our present study differ drastically from previous work done in both our field and in other related cases of online discussions. We found no correlation between participants’ professionalism and anonymity scores (rho = -0.004, p = 0.97). These results conflict with recent observational studies, which found negative effects of anonymity on professionalism both within the health professions [9, 22] and beyond [21]. One study found unprofessional content more commonly among medical students and physicians writing under pseudonyms compared to users writing under recorded names (p = 0.02) [9]. Another study conducted in an online community of practice for American soldiers found that eliminating the option to post anonymously reduced the negative comments by 89% [21]. Research on pharmacist bloggers found that unprofessional language was associated with users who blogged anonymously compared with those whose identity was disclosed [22]. While the ALiEM MEdIC online environment permitted users to post anonymously, the proportion of negative comments was much lower than that observed in these studies. No explicit instructions (e.g., code of conduct) or active restrictions were imposed on the participants with regards to the level of anonymity or the quality of comments.

There is a potential explanation for why our findings differed so dramatically from those in the literature. First, the preservation of a positive social reputation may be a more significant factor than concern for adverse repercussions in shaping participants’ online behavior [21]. Most participants in our study are linked to the FOAM community in some way and were likely to interact outside of the forum created by the blog [1]. The possibility that unprofessional comments in this forum could affect participants’ reputation in this online community as a whole may have inhibited unprofessional behavior.

Since the authors of this article are active members of the FOAM community, several speculations from experience can be made regarding that specific learning environment. The FOAM community can be viewed as a large, well-known virtual community of practice [23]. The association of ALiEM.com and this series with FOAM may have created an implied expectation for professional conduct that is in alignment with the greater FOAM culture. The moderators and active participants in our online discussions are viewed as core community members who welcome and guide new participants as they transition from passive readers to welcomed active participants. The ALiEM MEdIC moderators are trained to be professional and cordial participants who exemplify the expected behavior, perhaps creating implicit expectations for productive and respectful discussion [20]. Recall that, while the moderators were only usually rated a three on the professionalism scale, this was because they rarely contributed substantively to the discussion. Rather, they maintained their moderator role by politely greeting, engaging, and encouraging the other participants.  

Positive correlations were observed between a participant’s openness with their identity, the number of comments they made (p < 0.01), and cases they discussed (p < 0.05). These results also diverge from previous literature. An article that assessed the advantages and disadvantages of pseudonymity suggested that aliases may be a valuable asset to online learning for increasing participation and self-disclosure during group discussion [17]. They explained that pseudonyms create a context of ‘managed ambiguity’ by permitting relationships while selectively concealing or revealing elements of real-life identity. However, our findings reveal that this may not hold true in all contexts. We propose that if the discussion takes place in an environment that encourages mutual respect and professionalism, then aliases may not be required to promote active participation. This finding supports the notion that social media platforms in medical education must be critically examined to better understand their unique, underlying relationship structures [23]. The ALiEM MEdIC platform demonstrates the promise of professional online forums for educators that allow learners to engage with difficult issues while retaining their professional identities using new media.

### Limitations

The most significant limitation of this study was that it involved only a single online teaching environment and may not be generalizable to other contexts. However, this limitation was insurmountable as we are currently unaware of other online forums for medical professionals with similar characteristics that engage in online problem-based discussion.

Additionally, although we utilized previously derived scales for anonymity and professionalism, they had not previously been used in a medical education context. Operational definitions had to be modified to suit our online environment and medical education community.

Finally, very few remarks were scored as unprofessional and this may have resulted in spectrum bias [24]. There was also likely a ceiling effect (i.e., most of our scores were on the higher end of the scales) since the score proposed by Kilner and Hoadley has a maximum of four points, and many of our comments maxed out at four points. In Kilner and Hoadley’s original military online population, they found that up to 11% of comments were unprofessional. In our population, none (0%) of the comments scored were unprofessional, which is surprising based on previous literature [9, 21-22]. This may have resulted from an innate spectrum bias (i.e., because of the type of community, the standards set forth by the ALiEM website, and the website’s reputation), or because there were not enough comments to analyze (i.e., inadequate power). Since the previous literature has seen a higher incidence of unprofessionalism, we feel that it is more likely the former phenomenon at work here.

## Conclusions

The ALiEM MEdiC Series has effectively generated an online community that is both open about identity and professional. Expectations of professional and respectful behavior were exemplified through the group moderators, norms of the online community, reputation of the website, and objectives for each discussion. Further research is required to determine what factors encourage openness and professionalism online.
